# Aldosterone Antagonists in Monotherapy Are Protective against Streptozotocin-Induced Diabetic Nephropathy in Rats

**DOI:** 10.1371/journal.pone.0039938

**Published:** 2012-06-28

**Authors:** Nora F. Banki, Agota Ver, Laszlo J. Wagner, Adam Vannay, Peter Degrell, Agnes Prokai, Renata Gellai, Lilla Lenart, Dorottya-Nagy Szakal, Eva Kenesei, Klara Rosta, Gyorgy Reusz, Attila J. Szabo, Tivadar Tulassay, Chris Baylis, Andrea Fekete

**Affiliations:** 1 1st Department of Pediatrics, Semmelweis University, Budapest, Hungary; 2 SE-MTA “Lendulet” Diabetes Research Group, Hungarian Academy of Sciences and Semmelweis University, Budapest, Hungary; 3 Department of Medical Chemistry, Molecular Biology and Pathobiochemistry, Semmelweis University, Budapest, Hungary; 4 Department of Transplantation and Surgery, Semmelweis University, Budapest, Hungary; 5 Research Laboratory for Pediatrics and Nephrology, Hungarian Academy of Sciences and Semmelweis University, Budapest, Hungary; 6 2nd Department of Medicine and Nephrological Center, Pécs, Hungary; 7 Departments of Physiology and Functional Genomics and Medicine, University of Florida, Gainesville, Florida, United States of America; Pennington Biomedical Research Center, United States of America

## Abstract

Angiotensin converting enzyme inhibitors (ACEi) and angiotensin II receptor blockers (ARB) are the standard clinical therapy of diabetic nephropathy (DN), while aldosterone antagonists are only used as adjuncts. Previously in experimental DN we showed that Na/K ATPase (NKA) is mislocated and angiotensin II leads to superimposed renal progression. Here we investigated the monotherapeutic effect of aldosterone blockers on the progression of DN and renal NKA alteration in comparison to ACEi and ARBs. Streptozotocin-diabetic rats developing DN were treated with aldosterone antagonists; ACEi and ARB. Renal function, morphology, protein level and tubular localization of NKA were analyzed. To evaluate the effect of high glucose *per se;* HK-2 proximal tubular cells were cultured in normal or high concentration of glucose and treated with the same agents. Aldosterone antagonists were the most effective in ameliorating functional and structural kidney damage and they normalized diabetes induced bradycardia and weight loss. Aldosterone blockers also prevented hyperglycemia and diabetes induced increase in NKA protein level and enzyme mislocation. A monotherapy with aldosterone antagonists might be as, or more effective than ACEi or ARBs in the prevention of STZ-induced DN. Furthermore the alteration of the NKA could represent a novel pathophysiological feature of DN and might serve as an additional target of aldosterone blockers.

## Introduction

Diabetes mellitus is a complex metabolic disorder with nearly 170 million cases worldwide. The incidence is rapidly increasing and by the year of 2030 this number will almost double [1]. Diabetic nephropathy (DN) is the predominant cause of chronic kidney disease (CKD) and accounts for half of the end-stage kidney disease population [2]. Patients with DN also have abnormal lipoprotein metabolism and frequently develop severe atherosclerotic and cardiovascular complications resulting in a higher morbidity and mortality [3]. Since diabetes is a major drain on health and productivity-related resources for healthcare systems, the prevention and early treatment of DN would have enormous social and economical impact. Current therapeutic approaches based on the guidelines of the European and American Diabetes Associations still focus on angiotensin converting enzyme inhibitors (ACEi) and angiotensin II receptor blockers (ARB) [4], [5], while aldosterone antagonists are only used as adjuncts.

In diabetes the renin-angiotensin-aldosterone system (RAAS) is clearly activated [6]–[8], with increased renal angiotensin II (ANGII) and aldosterone activity. Renal angiotensinogen, angiotensin I and ANGII levels are approximately 1,000-fold greater as compared to their plasma levels [9]–[11]. Proximal tubules express angiotensinogen, renin, ACE, and ANGII receptors and facilitate even local aldosterone production [12] emphasizing the pivotal role of these cells in renal RAAS.

However glomerular, tubular and interstitial injuries are all characteristic for DN, alterations of renal RAAS significantly affect the tubules [13], [14]. Na/K ATPase (NKA) is the major force of sodium transport in proximal tubular cells, and as an ion transporter it is only active when inserted in its physiological place in the basal membrane [15]. In the kidney ANGII blocks this translocation of NKA leading to dysfunctional enzyme activity [16]. Recently we demonstrated also in streptozotocin (STZ)-diabetic rats that the renal NKA is mislocated from the tubular basal membrane toward the cytoplasm and thus becomes non-functional. Exogenous ANGII administration led to further impairment of NKA and superimposed progression of DN [17], [18].

Our aim in the present study was to characterize the monotherapeutic effect of different aldosterone antagonists in comparison to other RAAS inhibitors in the pathophysiology of DN and to investigate the role of NKA. Since both hyperglycemia and hyperosmolarity are pathological features of diabetes *in vivo*, we also investigated the direct effects of hyperglycemia on tubular cells *in vitro.*

## Results

### Aldosterone antagonists ameliorated all metabolic and renal parameters in STZ-induced diabetic rats

Metabolic and renal parameters are summarized in Table 1. After 7 weeks of diabetes rats had developed lower body weight and higher blood glucose level than controls. Serum total cholesterol, LDL-cholesterol and triglyceride levels were higher in diabetic rats as compared to controls. Kidney weight to body weight ratio, serum creatinine, BUN, potassium and protein to creatinine ratio values were increased, while serum sodium level was lower in diabetic rats suggesting the presence of renal hypertrophy and impaired kidney function.

**Table 1:** Metabolic and renal parameters of control, diabetic and treated diabetic rats.

Parameter	Control	Diabetic (D)	D+Enalapril	D+Losartan	D+Spironolactone	D+Eplerenone
Body Weight (g)	342±13	260±29^***^	256±42	250±65	349±29^§§§^	273±55
Blood Glucose (mmol/L)	11.6±1.1	43.6±3.2^***^	35.6±5.2^§§^	36.1±5.9^§§^	33.2±2.4^§§§^	38.5±4^§§^
Se-Cholesterol (mmol/L)	1.72±0.43	4.1±2.3^*^	3.13±1.64	2.72±0.91	1.64±0.32^§§^	2.28±0.44^§^
Se-LDL-Cholesterol (mmol/L)	UD	1.63±1.95^*^	0.65±1	0.48±0.54	UD^§^	UD^§^
Se-Triglyceride (mmol/L)	1.32±0.17	4.94±3.7^*^	4.54±5.17	2.45±1.51	0.79±0.29^§§^	2.16±1.17^§^
Kidney/body weight x 100	0.42±0.02	0.67±0.06^***^	0.53±0.1^§§^	0.54±0.07^§§^	0.56±0.07^§§^	0.58±0.05^§§^
Se-Creatinine (μmol/L)	55.6±5.4	64.6±6.7^*^	57.8±9.8	54.8±7.1^§§^	56.7±4.5^§§^	67.2±5.8
Blood Urea Nitrogen (mmol/L)	7.12±0.28	15±2.8^**^	11.9±2.4	11.9±1.5	8.61±1.05^§§^	11.4±2.3^§^
Se-Potassium (mmol/L)	5.78±0.43	7.26±0.86^**^	7.24±1.08	6±0.5^§^	5.34±1.39^§§^	6.12±0.84^§^
Se-Sodium (mmol/L)	154±8.46	135±3.7^***^	137.2±3.1	138±1	141±2^§§^	140±2.3^§§^
Urinary protein/creatinine	0.12±0.02	1.77±0.89^**^	0.14±0.03^§§§^	0.21±0.1^§§§^	0.35±0.1^§§^	0.04±0.02^§§^

Data are means ± SD, n = 8–10/group, ^*^p<0.05 vs. C; ^§^p<0.05 vs. D, ^**^p<0.01 vs. C; ^§§^p<0.01 vs. D, ^***^p<0.001 vs. C; ^§§§^p<0.001 vs. D; respectively. UD–undetectable.

Only Spironolactone prevented weight loss of diabetic animals, while blood glucose level was lower in all RAAS blocker treated animals but not normalized. Aldosterone antagonists ameliorated each lipid profile parameter, while Enalapril and Losartan had no effect. Parameters representing kidney function were affected as shown in Table 1: Spironolactone ameliorated all parameters investigated and Eplerenone was also very effective but failed to preserve serum creatinine.

### Aldosterone blockers attenuated the structural lesions of DN

**Figure 1:**
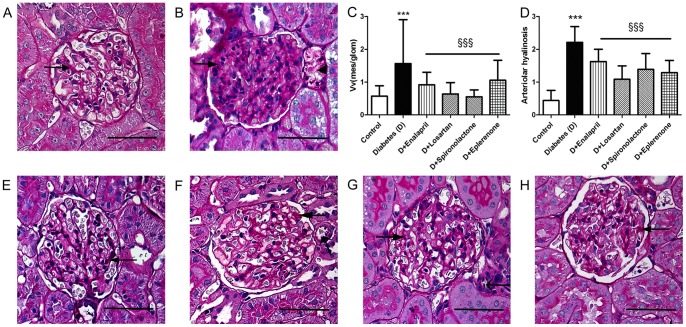
Renal histopathology in control, diabetic and treated diabetic rats. Aldosterone antagonists were the most effective in attenuating the structural lesions of DN. Representative PAS staining of kidney sections (40x magnification; scale bar –50 μm): non-diabetic control (A), STZ-induced diabetic (B), Enalapril (E), Losartan (F), Spironolactone (G) and Eplerenone (H) treated diabetic rats (n = 8–10/group). Long, wide headed arrows point on mesangial matrix; long, narrow headed arrows on arterioles. Armanni-Ebstein lesions are marked with short, wide headed arrows. *C*: Mesangial fractional volume values (Vv) are defined by the ratio of mesangial area/glomerular tuft area. The mesangial area is determined by assessment of PAS-positive and nucleus-free areas in the mesangium. *p<0.05 *vs* Control; §p<0.05 *vs* Diabetes, respectively; (bars show means ± SD). *D*: Arteriolar hyalinosis is defined by the average of hyalinized quarters of arterioles. The hyalin is determined by assessment of PAS-positive and nucleus-free areas within the arterioles. *p<0.05 *vs* Control; §p<0.05 *vs* Diabetes, respectively; (bars show means ± SD).

The evaluation of DN was based on glomerular lesions (increased PAS positive area: mesangial matrix expansion and basal membrane thickening) with a separate assessment of arteriolar hyalinosis and tubular atrophy (Armanni-Ebstein lesion). Control kidneys showed normal glomerular structure, no tubular lesions and minimal arteriolar hyalinosis (Fig. 1/A). Kidneys of STZ-induced diabetic rats developed severe mesangial matrix expansion and obliteration of capillaries with regional adhesion of the glomerular tuft to Bowman's capsule at the site of mesangial matrix expansion (Fig. 1/B). These alterations were accompanied by arteriolar hyalinosis exceeding the mean area of capillary lumen. The tubules were dilated and lined with flattened epithelium. Armanni-Ebstein lesions were observed in several tubules with typical deposits of glycogen.

Mesangial fractional volume value was the lowest in D+Spironolactone but it was also decreased in the other treatment groups (Fig. 1/C). Aldosterone antagonists were also effective in minimizing arteriolar hyalinosis and the presence of Armanni-Ebstein anomalies (Fig. 1/D–H).

### Diabetes and hyperglycemia elevated tubular NKA protein level

**Figure 2:**
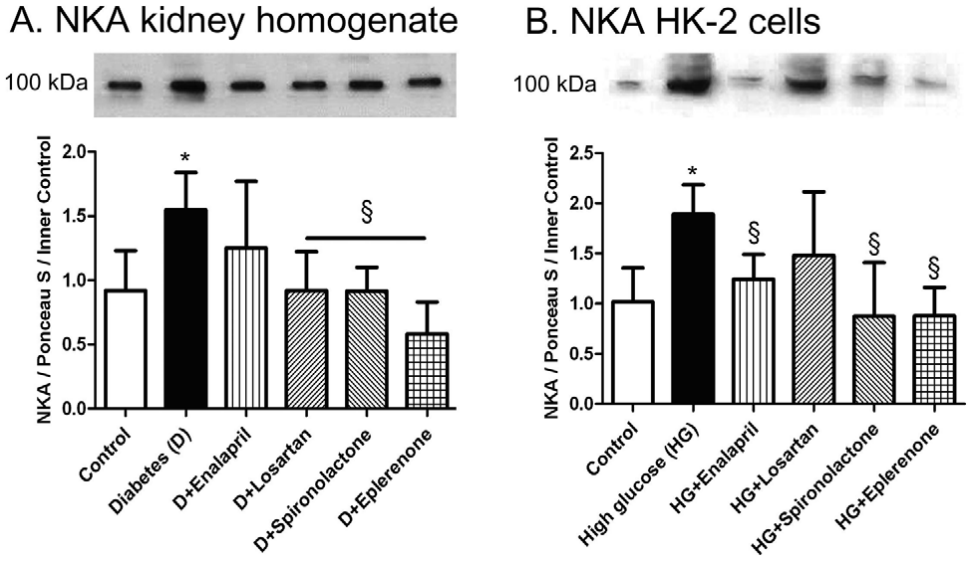
Western blot analysis of Na/K ATPase (NKA). Aldosterone antagonists were the most effective in decreasing diabetes and hyperglycemia induced elevation of tubular NKA protein level. Top panel: Representative examples of Western blot analysis. Lower panels: *A*: Densitometric analysis of NKA protein levels in kidney homogenates of control, diabetic and treated diabetic rats. *B*: Densitometric analysis of NKA protein levels in HK-2 tubular cells. Bar graph represents densitometric analysis from multiple experiments. Data represent means ± SD; *p<0.05 *vs* Control; §p<0.05 *vs* Diabetes, respectively; (bars show means±SD; n = 8–10/group). IOD – integrated optical density.

NKA protein level was almost doubled both in kidney homogenates of STZ-diabetic rats (Fig. 2/A) and hyperglycemic (35 mM) tubular cells (Fig. 2/B) compared to controls, while aldosterone antagonists were the most effective in decreasing this elevated level of NKA (Fig. 2/A–B).

A similar change in osmolarity obtained by the use of 30 mM mannitol+5 mM glucose failed to reproduce these effects in tubular cells (data not shown).

### Aldosterone inhibitors prevented the mislocation of NKA induced by diabetes in proximal tubules

**Figure 3:**
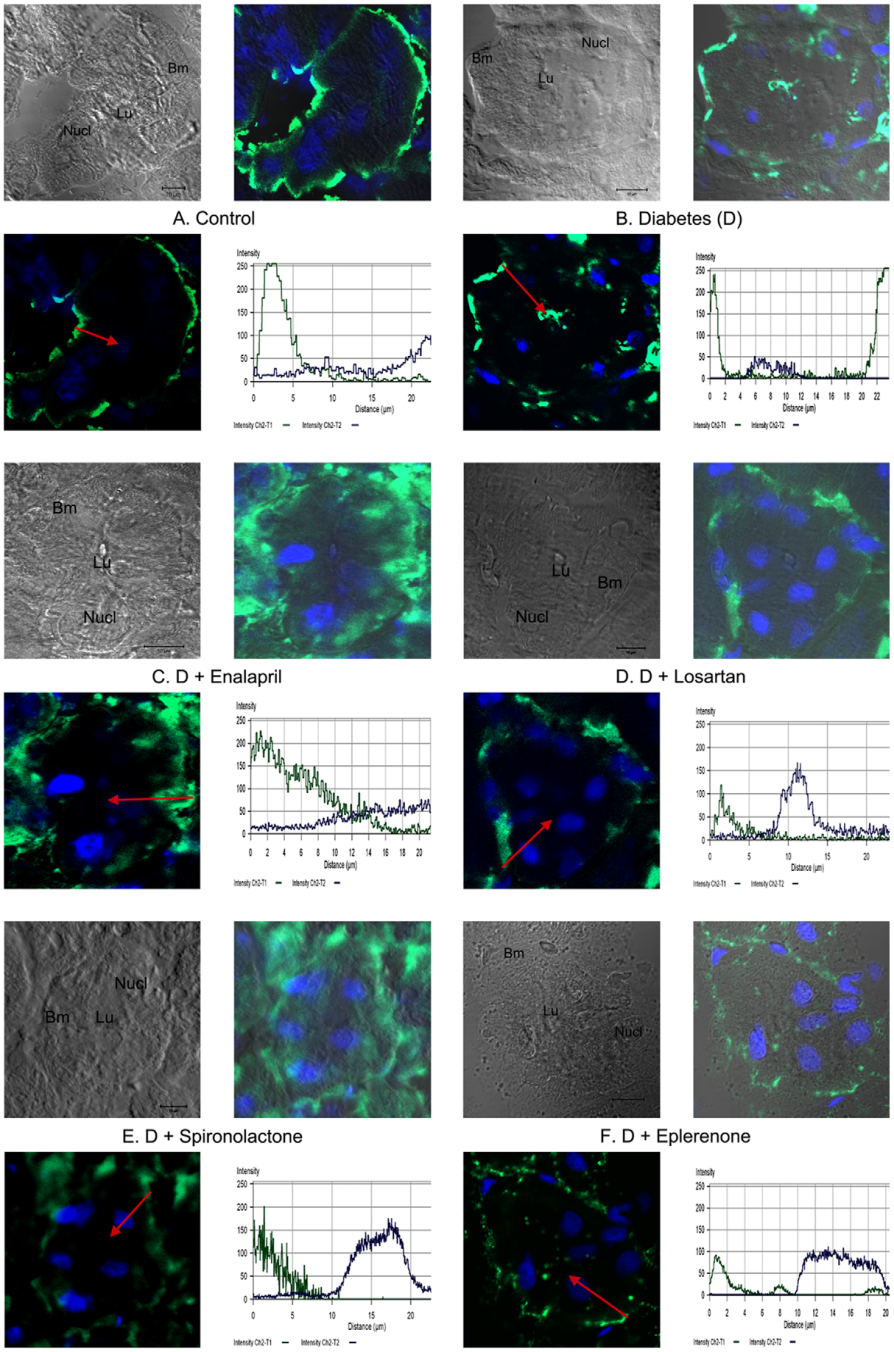
Confocal images of control, diabetic and treated diabetic rats. Aldosterone inhibitors prevented the mislocation of NKA induced by diabetes in proximal tubules. Representative pictures of immunofluorescence staining of kidney sections for Na/K ATPase (NKA, green) in control (A), streptozotocin-diabetic (B) and diabetic, Enalapril (C), Losartan (D), Spironolactone (E) and Eplerenone (F) treated rats (63x magnification; scale bar–10 μm). Nuclei are stained blue with Hoechst. PT-proximal tubule, DT-distal tubule, Bm-basal membrane, Lu – apical membrane at the lumen and Nucl – nuclei. Fluorescent signal intensity of NKA (green) generated from a line shown as red arrow in the merged image are shown on the bottom right of each panel.

NKA distribution showed a linear, basolateral membrane associated pattern in control animals which was changed to a cytoplasmic or even to an apical membrane associated staining in diabetic animals (Fig. 3/A–B). Aldosterone antagonists prevented this mislocation the most, although the linear staining pattern of NKA was slightly widened (Fig. 3/C–F).

### Aldosterone antagonists restored heart rate in STZ-induced diabetic rats, while neither diabetes, nor RAAS blockers influenced MAP

Arterial blood pressure and heart rate were monitored by the non-invasive tail cuff method. Heart rate was lower in diabetic animals, but was restored to the level of controls by aldosterone antagonists (Table 2). MAP remained unchanged after 7 weeks of untreated diabetes and after the treatment with each RAAS inhibitor (Table 2), reflecting the non-depressor dose of the substances.

**Table 2:** Mean arterial blood pressure (MAP) and heart rate of control, diabetic and treated diabetic rats.

Parameters		Control	Diabetic (D)	D+Enalapril	D+Losartan	D+Spironolactone	D+Eplerenone
**MAP** **(mmHg)**	*Before Treatment*	105±17	117±20	103±33	110±18	118±22	118±25
	*After Treatment*	100±14	103±21	116±29	109±17	108±19	125±11
**Heart Rate (bpm)**	*Before Treatment*	443±48	366±47*	346±41	334±39	349±32	366±42
	*After Treatment*	422±70	350±49*	356±26	362±43	400±20§	389±35§

Data are means ± SD, n = 8–10/group, *p<0.05 vs. Control; §p<0.05 vs. Diabetes, respectively. Bpm-beat/min.

## Discussion

Since chronic kidney disease due to DN is becoming an ever larger health burden worldwide more effective therapies are desperately needed. In the past years ACEi and ARB have become the standard care for diabetic patients with microalbuminuria [5], however increasing evidence suggests that these agents do not slow the progression of DN significantly [19], [20]. In DN aldosterone antagonists are still underused since Spironolactone is applied occasionally as an adjunctive therapy while Eplerenone is not licensed yet.

Therefore the primary goal of our study was to evaluate the monotherapeutic efficacy of different aldosterone antagonists in comparison to ACEi and ARB in the protection against DN. According to our results aldosterone antagonism both by Spironolactone or Eplerenone might be a valuable choice to slow the progression of DN.

Hyperkalemia poses a therapeutic dilemma for the treatment with aldosterone antagonists, especially in diabetic patients. However in the recent years several randomized-well controlled trials showed that in case of monotherapy the incidence of significant hyperkalemia is relatively low [21], [22]. Although we neither found elevated potassium levels in the aldosterone-antagonists treated group, according to the literature special precaution is needed in combination therapy of aldosterone antagonist with other RAAS blockers, especially in diabetic patients since diabetes is an independent risk factor for hyperkalemia [23].

It has been already suggested that antihypertensive treatment by different RAAS blockers provide renoprotection independent of blood pressure lowering. Izuhara et al showed that beyond decreasing blood pressure the unique renoprotective properties of ARB olmesartan are also related to other factors (e.g. decreased oxidative stress, etc) [24]. To test whether this renoprotection of RAAS blockade is limited to antihypertensive doses, or is also seen with lower dosages we chose treatment protocols avoiding blood pressure changes but remaining effective in blocking ACE [25], ANGII receptor 1 [26] or aldosterone [27].

In the present study neither diabetes nor RAAS blockers changed blood pressure, which confirms the non-depressor dose of our protocols. However tachycardia is a well-known feature of diabetic patients [28]; diabetic rats have proven resting bradycardia [29], [30], due to the dysfunction of both the sympathetic and parasympathetic innervation of the baroreflex [30]. Here only aldosterone antagonists restored lower heart rates of diabetic animals back to the level of controls. This effect of Spironolactone and Eplerenone could be partly explained by the prevention of baroreceptor and baroreflex depression via inhibiting the aldosterone induced increase of NKA synthesis and activity in the carotid sinus [31].

In line with previous data [17], [32] in the present study untreated diabetic rats had nearly 25 % lower body weight than controls and this was prevented by Spironolactone, but not by Eplerenone, ACEi or ARB. Previously it has been shown that after STZ treatment body weight of male rats is reduced compared to control males; but this has been not observed among females [33]. Since Spironolactone has lower stronger anti-androgenic property (due to the lack of the 9,11-epoxide group) than Eplerenone [32]; we hypothesize that Spironolactione might be more effective on the account of this phenomenon.

In the present study aldosterone inhibitors reduced the elevated blood glucose level of diabetic animals. Although STZ injection leads to the destruction of pancreatic ß-cells, a residual insulin activity still exists even after 6 weeks [34]. Since aldosterone impairs insulin signaling, it is conceivable that Spironolactone and Eplerenone might be effective through inhibiting aldosterone induced insulin resistance [32].

In diabetic patients altered lipoprotein metabolism [3] and an abnormal lipid profile [35] contribute to accelerated atherosclerosis and increased risk of cardiovascular disease [36]. Parallel to other animal studies [37], we also detected remarkably elevated total and LDL-cholesterol and triglyceride levels in diabetic rats. Aldosterone antagonists improved all lipid parameters, while ACEi and ARB had no effect. Spironolactone has been already shown to ameliorate serum lipid parameters [32], but we are the first to report that Eplerenone is equally effective. Aldosterone antagonists might exert their beneficial effect partly by decreasing insulin resistance in the liver [38]. However, it is also conceivable that the lipid lowering affinity of aldosterone antagonists in diabetes is provided by inhibiting proinflammatory cytokine production from white adipose tissue as well [39].

In our study the impaired renal function and increased kidney to body weight ratio of diabetic animals hints at the toxic effect of glucose and suggests renal damage. Histological hallmarks of DN including mesangial matrix expansion, arteriolar hyalinosis and Armanni-Ebstein lesions were also present in diabetic rats. Armanni-Ebstein lesions – the vacuolarization of tubular epithelia – are caused by aggregated glycogen as a result of increased tubular glucose uptake. The ability of the proximal tubuli to reabsorb glucose is amplified as the filtered load is increased due to the elevation in plasma glucose. In the present study aldosterone blockade was the most effective in improving kidney function and reducing renal structural damage. Since after aldosterone antagonist treatment blood glucose level was lower as well, one might hypothesize that in these groups the reduced tubular glucose load could lead to milder glucotoxicity-related kidney damage.

A Na^+^ gradient is required for the ongoing tubular transport of glucose, which is created by the basolaterally located NKA [40]. In diabetes NKA plays a role in the development of impaired renal glucose and Na^+^ handling and in loss of renal function. However it has already been demonstrated that NKA function is influenced by ANGII inhibitors, in diabetes data are scarce with one study reporting that ACEi prevents the increase of NKA in the diabetic retina [41].

Previously in STZ-diabetic rats, we demonstrated that renal NKA is elevated; the enzyme is mislocated from the tubular basal membrane to the cytosol and becomes non-functional. This in line with recent findings of Galuska et al showing that hyperglycemia induces the mislocation of NKA from the basolateral membrane to the cytosol in human tubular cell culture [42]. We also showed that ANGII administration exerts similar changes, while ANGII treatment in STZ-diabetes has a superimposed effect leading to pronounced renal damage and NKA alteration [17]. Here we extended our findings by showing that ACEi and ARB decreases diabetes-induced NKA elevation and prevents enzyme mislocation. Furthermore we demonstrated that aldosterone blockade is even more effective in preventing these diabetic NKA alterations than ACEi or ARB tretament. We confirmed these results also *in vitro*, and showed that the changes in NKA are likely to be due to the presence of hyperglycemia than to glucose-induced hyperosmolarity.

According to our results a monotherapy with aldosterone antagonists might be as, or more effective in the prevention of STZ-induced DN, compared to ACEi or ARB. Moreover the alteration of NKA could represent a novel pathophysiological feature of DN and might serve as an additional target of RAAS blockers. In summary our results might facilitate the monotherapeutic application of Spironolactone and might open new perspectives for Eplerenone in the clinical management of DN, however well-controlled human clinical trials are needed to confirm these suggestions.

## Materials and Methods

### Ethic Statement

All animal procedures were approved by the Committee on the Care and Use of Laboratory Animals of the Council on Animal Care at the Semmelweis University of Budapest, Hungary (TUKEB 99/94).

### Study design, Induction of Diabetes and Experimental Groups

All substances were purchased from Sigma-Aldrich Ltd. (Budapest, Hungary). Diabetes was induced in male Wistar rats (175–200 g, 8 weeks) by 65 mg/kg STZ *i.v.* (dissolved in 0.1 M citrate buffer; pH 4.5). Animals were considered diabetic if blood glucose concentrations increased to ≥15 mmol/L within 72 h after STZ injection and remained elevated. Five weeks after the induction of diabetes, animals were randomly divided into five groups and received by oral gavage (n = 8–10/group): vehicle (D); ACEi Enalapril (D+Enalapril, 40 mg kg-1 day-1); ARB Losartan (D+Losartan, 20 mg kg-1 day-1); the non-selective aldosterone antagonist Spironolactone (D+Spironolactone, 50 mg kg-1 day-1) or the selective aldosterone antagonist Eplerenone (D+Eplerenone 50 mg kg-1 day-1). As a result of the presence of a 9,11-epoxide group in the structure of Eplerenone, its selectivity for the aldosterone receptor is enhanced and its affinity for the progesterone and androgen receptors is very low (i.e.<1 % and<0.1 %, respectively, of the receptor binding of Spironolactone). The doses were selected and adopted from previous studies where effective blockade of ACE [25], ANGII receptor [26] and aldosterone activity [27] was observed without leading to changes of systemic blood pressure. Non-diabetic age-matched control animals were injected with citrate buffer and sacrificed after 7 weeks (n = 8–10/group).

After 2 weeks of treatment with ACEi, ARB or aldosterone antagonists rats were anesthesized, blood and urinary samples were collected and the kidneys were removed, weighed and a section fixed for histology and the remained immediately snap-frozen for further investigations.

### Measurement of arterial blood pressure

Arterial blood pressure (systolic, diastolic, mean (MAP)) and heart rate were monitored from the tail artery with CODA Standard monitor system (EMKA TECHNOLOGIES, Paris, France), which uses proprietary volume pressure recording, a clinically validated technology providing significant correlation with telemetry [43].

### Measurement of metabolic and renal parameters

Body and kidney weight were measured, kidney/body weight ratio was calculated. Serum metabolic (glucose, total and HDL-cholesterol, triglycerides) and renal functional parameters (sodium, potassium, creatinine and BUN) were photometrically determined with commercially available kits on a Hitachi 912 photometric chemistry analyzer. Urinary protein to creatinine ratio was also calculated.

### Renal histology and morphometric analysis

Kidney was fixed in 10 % formalin, paraffin embedded, 5 µm wide sections were taken and stained with periodic acid-Schiff (PAS) for determination of glomerular matrix expansion, vascular hyalinosis and tubulointerstitial lesions as previously described [44]. Briefly, glomerular hypertrophy was determined by measuring the glomerular tuft area of 50 glomerular cross-sections excluding incomplete glomeruli along the sample edge. Hyaline was determined by assessment of PAS-positive and nucleus-free areas within the arterioles. Arteriolar hyalinosis was defined by the average of hyalinized quarters of arterioles. The presence of Armanni-Ebstein lesions was also evaluated. The analysis was performed on a double blinded fashion with computer-assisted morphometry using AxioVision 4.8 software on a Zeiss AxioImager A1 light-microscope.

### Western blot analysis

All reagents for PAGE and Western blot were purchased from Sigma-Aldrich Ltd. Kidney samples were sonicated and resuspended in lysis buffer. Protein concentration estimations were performed with a detergent-compatible protein assay kit (Bio-Rad Hungary, Budapest, Hungary). Samples containing 50 μg total protein were electrophoretically resolved on a 10 % polyacrylamide gel and transferred to nitrocellulose membranes. The membranes were incubated with the specific mouse NKA-α1 subunit (1∶1,000, Santa Cruz Biotechnology, Budapest, Hungary) antibody. After repeated washing the blots were incubated with the corresponding goat anti-mouse (1∶12,000, Santa Cruz Biotechnology) antibody. Non-diabetic rat brain cytosol was used as a positive control.

Bands of interest were detected using enhanced chemiluminescence detection (ECL; GE Healthcare Life Sciences, Budapest, Hungary) and quantified by densitometry (Versadoc, Quantity One Analysis software; Bio-Rad Hungary) as integrated optical density (IOD) after subtraction of background. The IOD was factored for Ponceau red staining to correct for any variations in total protein loading and for internal control (rat brain). The protein abundance was represented as IOD/Ponceau S/Internal control.

### Fluorescent immunohistochemistry

Frozen kidney sections were embedded in Shandon cryomatrix (Thermo Fisher Scientific) and cut to 5 µm slides with a cryostat. Samples were incubated for one hour with the specific mouse NKA-α1 (1∶200, Santa Cruz Biotechnology) antibody. After repeated washing slides were incubated with goat anti-mouse Alexa Fluor 488 conjugate and counterstained with Hoechst 33342 (Sigma-Aldrich Ltd.) to visualize nuclei. Appropriate controls were performed omitting the primary antibody to assure the specificity and to avoid autofluorescence. Sections were analyzed with a Zeiss LSM 510 Meta confocal laser scanning microscope with objectives of 20x and 63x magnification.

### Statistical analysis

Results were expressed as means ± SD. Statistical analyzes were performed using Prism software (version 5.00; GraphPad Prism Software, GraphPad Software, Inc., La Jolla, California, USA). Multiple comparisons and possible interactions were evaluated by one-way ANOVA followed by Fisher's protected least significant difference test. For non-parametrical data the Kruskal-Wallis ANOVA on ranks was used. *P* values of<0.05 were considered significant.
